# Systematic quantitative evaluation of Plan-IQ for intensity-modulated radiation therapy after modified radical mastectomy

**DOI:** 10.1038/s41598-021-01305-3

**Published:** 2021-11-08

**Authors:** Kunzhi Chen, Zhuangzhuang Zheng, Lijuan Ding, Na Tao, Libo Wang, Wenming Xia, Huidong Wang, Xin Jiang

**Affiliations:** 1 Department of Radiation Oncology, The First Hospital of Jilin University, 71 Xinmin Street, Changchun, 130021 China; 2 Jilin Provincial Key Laboratory of Radiation Oncology & Therapy, Changchun, 130021 China; 3 NHC Key Laboratory of Radiobiology, School of Public, Health of Jilin University, Changchun, 130021 China; 4 Department of Pediatric Surgery, The First Hospital of Jilin University, Changchun, 130021 China

**Keywords:** Cancer, Physics

## Abstract

Radiotherapy (RT) is one of the main treatment strategies of breast cancer. It is challenging to design RT plans that can completely cover the target area while protecting organs at risk (OAR). The Plan-IQ feasibility tool can estimate the best sparing dose of OAR before optimizing the Plan. A systematic quantitative evaluation of the quality change of intensity-modulated radiation therapy (IMRT) using the Plan-IQ feasibility tool was performed for modified radical mastectomy in this study. We selected 50 patients with breast cancer treated with IMRT. All patients received the same dose in the planning target volume (PTV). The plans are categorized into two groups, with each patient having one plan in each group: the clinically accepted normal plan group (NP group) and the repeat plan group (RP group). An automated planning strategy was generated using a Plan-IQ feasibility dose volume histogram (FDVH) in RP group. These plans were assessed according to the dosimetry parameters. A detailed scoring strategy was based on the RTOG9804 report and 2018 National Comprehensive Cancer Network guidelines, combined with clinical experience. PTV coverage in both groups was achieved at 100% of the prescribed dose. Except for the thyroid coverage, the dose limit of organs at risk (OAR) in RP group was significantly better than that in NP group. In the scoring analysis, the total scores of RP group decreased compared to that of NP group (P < 0.05), and the individual scores of PTV and OAR significantly changed. PTV scores in RP group decreased (P < 0.01); however, OAR scores improved (P < 0.01). The Plan-IQ FDVH was useful for evaluating a class solution for IMRT planning. Plan-IQ can automatically help physicians design the best OAR protection plan, which sacrifices part of PTV, but still meets clinical requirements.

## Introduction

Breast cancer is a common malignancy in women, accounting for approximately 30% of new cancer cases in all women in the United States^1^. Modified radical mastectomy is the primary treatment for patients with breast cancer. Radiotherapy (RT) after a radical mastectomy effectively increases the local control rate and reduces the mortality of patients with breast cancer^2–5^. According to the recommendations of the National Comprehensive Cancer Network (NCCN) and Radiation Therapy Oncology Group (RTOG), patients with high risks of local recurrence need RT after mastectomy.

Intensity-modulated radiation therapy (IMRT) and volumetric modulated arc radiotherapy (VMAT) have been widely used to treat malignancies. IMRT can improve the tumor control rate and patient survival by increasing the dose in the target area and reducing the dose to organs at risk (OAR)^6–8^. Many studies on IMRT after modified radical mastectomy have benefited many patients^9,10^. When designing the RT plan, we sought to minimize the dose to the normal tissues and optimize the prescribed dose coverage, homogeneity, and conformal degree of the area (planning target volume (PTV)). However, the process of dose optimization using IMRT is affected by several factors, which results in a significant difference in the quality of regimens^11^. Most steps involved in generating the anatomical structure and optimizing the treatment plan can be automated. The use of automatic planning can reduce human variability to achieve predefined target dose goals.

Plan-IQ feasibility (Beijing HGPT Technology & Trade Corporation, China) is a tool that can estimate the best sparing dose of OAR before optimizing the plan^12^. Plan-IQ can predict OAR dose using a model that takes a 3-dimension dose clouds built outside targets as a benchmark to reflect the properties of radiation distribution observed in the media, which is computed using a series of energy-specific dose spread calculations^13–15^. For patients, the calculation is performed on the heterogeneous dataset, taking into account the high- (penumbra driven) and low- (percentage depth dose and scatter-driven) gradient dose spreading. A “best possible sparing” feasibility dose volume histogram (FDVH) for an OAR will be produced based on the benchmark dose, and estimate more easily achievable FDVH curves^15^. Its automated optimization process can reduce level differences among physicians, which cannot be achieved using dose volume histogram parameters (DVH). In this study, Plan-IQ was used to guide the modification of the organ dose optimization limit of IMRT. The systematic quantification of FDVH was used to describe the changes in plan-IQ on the quality of IMRT plans.

## Materials and methods

### Patients

Female patients with breast cancer who received IMRT were selected from our department between January 2018 to August 2020. Fifty patients received preventive RT with PTV; the prescribed dose was 50 Gy in 25 fractions. This study is a retrospective study, informed consent was exempted because the study did not harm patients by the Ethics Committee of the First Hospital of Jilin University. There are not conflicting interests with the other ethical parameters**.**

### Image data and position fixation

Patients were placed on a CIVICO carbon fiber RT bed. They were placed according to the following methods: (1) the patients were placed supine; (2) the patients’ head was supported by a B to E Styrofoam soft pillow and tilted slightly toward the healthy side; (3) both arms were placed on the special fixing bracket, and both hands were placed on the forehead; (4) the irradiation area was covered with 1.0 cm equivalent tissue filler and fixed with thermally shrinkable peritoneum; and a 24-row spiral Siemens computer tomography (CT) was used to scan patients from the mandible to the navel during normal breathing with a slice thickness of 5 mm and a plane voxel size of 1 mm × 1 mm, and then uploaded to the Philips Pinnacle@9.10 treatment planning system for 3D image reconstruction. The CT image was uploaded to the dedicated RT server of Varian Eclipse TPS 13.5 (Radiotherapy Treatment Planning System) to design the RT plan.

### Delineation of RT targets and OAR

According to the NCCN guidelines 2018 and report No.9804 of RTOG, the clinical target volume (CTV) and relevant OAR were contoured by qualified radiation oncologists:

1. Chest wall target: The upper boundary was located at the edge of the collar bone head down to 1 cm, connecting with the supraclavicular field. The lower boundary was 2 cm below the undamaged breast plica. The anterior boundary included the skin of the chest wall. The posterior boundary included the ribs and intercostal muscles. The inner bound was the midline of the body. The lateral border was the midaxillary line.
2. Target of supraclavicular lymphatic drainage: The upper boundary was the level of the cricothyroid membrane. The lower boundary was 1 cm below the lower margin of the clavicle head and connected to the chest wall. The inner boundary was the inner margin of the sternocleidomastoid, descending to the midpoint of the sternal notch. The outer boundary extended to the humeral head. Five millimeters were added to the three-dimensional margin of the CTV to obtain PTV.
3. OAR: ipsilateral lung, heart, thyroid, spinal cord, shoulder joint-R, breast-R, esophagus, trachea, and intestines.

### Prescription dose of PTV and dose limitation for OAR

The planning objectives for PTV were the relative volume that received ≥ 100% of the prescribed dose > 95%, and the maximum point dose was < 110% of the prescribed dose. The dose coverage and homogeneity of PTV were assessed based on the dose distribution, DVH, and the trade-off between the dose delivered to PTV and OAR sparing. The planning objectives for OARs were as follows: ipsilateral lung, receiving more than 20 Gy (V_20_) < 20%, receiving more than 10 Gy (V_10_) < 25%, receiving more than 5 Gy (V_5_) < 35%; volume of heart receiving more than 40 Gy (V_40_) < 30% and that receiving more than 30 Gy (V_30_) < 40%; mean dose of heart (D_mean_) < 10 Gy; breast-R: a dose of 1 cubic centimeter volume (D_1cc_) < 5 Gy.

### Planning design

The plans are categorized into two groups, with each patient having one plan in each group: the clinically accepted normal plan group (NP group) and the repeat plan group (RP group). An automated planning strategy was generated in RP group using a Plan-IQ FDVH, which is a radiotherapy planning analysis software. Both NP and RP groups were subjected to fixed fields irradiation. For each enrolled patient, the layout of the beam, the dose prescription of PTV, and the initial optimization parameters of each OAR were set by loading a predefined technical script during the planning process. The lower bound part of the target was illuminated by penetrating irradiation with four supplementary upper oblique angles, whereas the upper part was illuminated by 5-fields and half-field irradiation.

### Plan evaluation

To comprehensively evaluate the changes in PTV and OAR dosimetry parameters and evaluate the quality of IMRT plans, we set up a new plan quality metric (PQM)^11^, which was defined according to the existing PQM, RTOG 9804 report, NCCN guidelines 2018, and our clinical experience (Supplement Table S1).

Target dosimetry parameters included the volume reaching the prescribed dose (V_RX_), homogeneity index (HI), and conformal index (CI). V_RX_ = PTV volume (cc) covered by specified dose (50 Gy)/ total PTV volume. HI = D_1_ / D_99_, where D_1_ is the dose received by 1% of the target volume, and the rest by analogy. CI = (Vt, ref/Vt) × (Vt, ref/Vref), where Vt is the volume of PTV, Vt. ref is the volume of PTV wrapped around the isodose curve of the prescription dose (50 Gy), and Vref is the volume of all areas wrapped around the isodose curve of the prescription dose (50 Gy). The closer the CI is to 1, and the HI value is to 0, the better the uniformity and conformality of PTV. OAR dose assessment included D_mean_ of the heart, D_max_ (maximum dose in the target area) of healthy breast, average dose (V_5_, V_10_, V_20_, D_mean_) of the affected lung, D_mean_ and D_max_ of the humeral head-L, and thyroid and monitor unit (MU).

### Statistical analysis

The experimental data in this study were collated using Microsoft office and statistically analyzed using PASW Statistics 22 and SPSS 22.0. The measurement data are expressed as the mean ± standard deviation (SD). If the comparison between NP and RP groups conformed to a normal distribution, the paired t-test was used, whereas the non-parametric test was used if the comparison did not accord with normal distribution. The significance level was set at *P* < 0.05.

### Ethics approval and consent to participate

All experimental protocols were approved by the Ethics Committee of the First Hospital of Jilin University. This study is a retrospective study, all patients have received a standard accepted normal plan. An automated planning strategy was generated using a Plan-IQ after the treatment. There were no any harm to patients, so the informed consent was remission.

### Consent for publication

All authors agree to publish the article.

## Results

### The dose distribution in the target area

**Figure 1:**
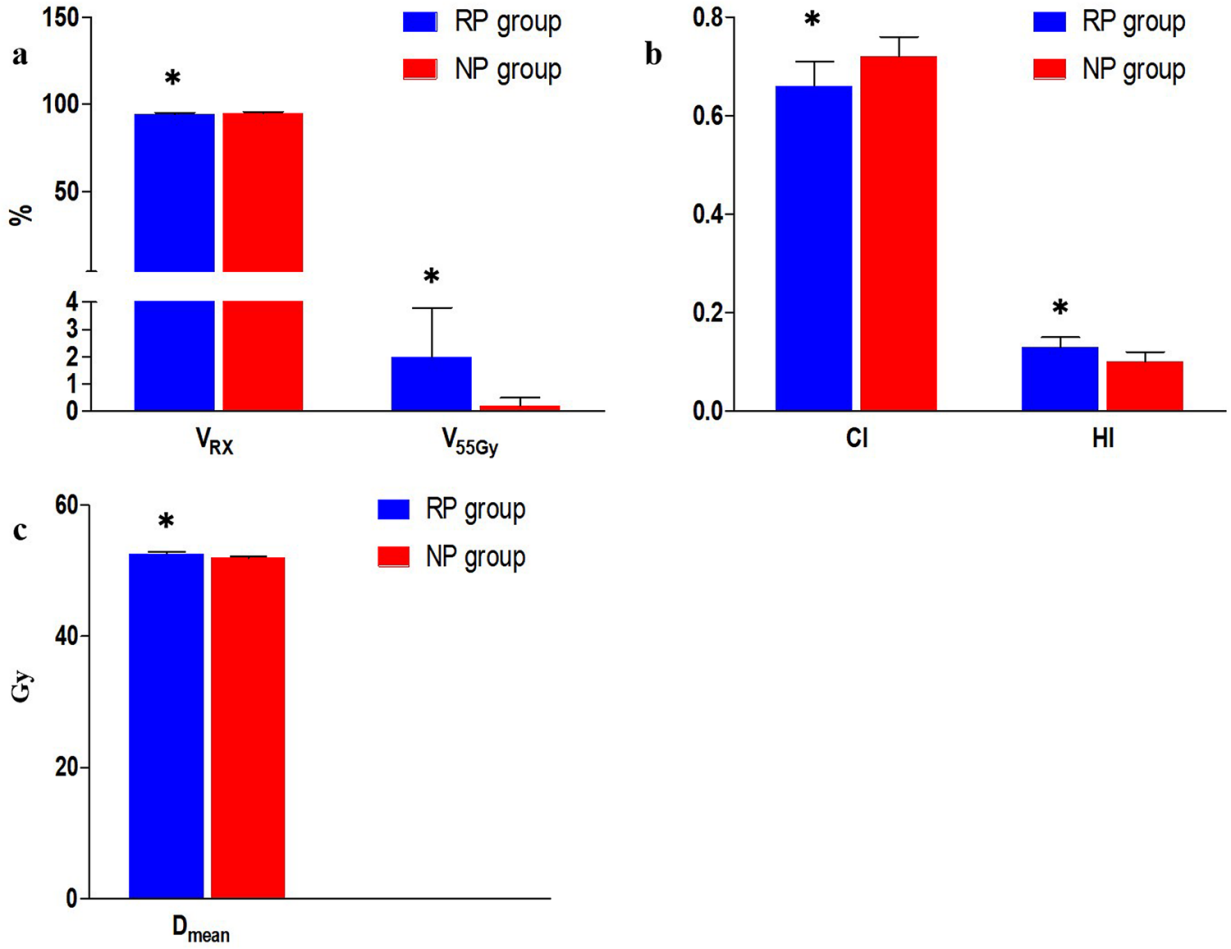
Dosimetry comparison for the PTV of RP group and NP group. VRX of the NP group was higher than that of the RP group (t = − 4.57, P < 0.01) (**a**). CI and HI of NP group was higher than that of RP group (t = − 12.99, P < 0.01; t = − 6.14, P < 0.01) (**b**). V55 and Dmean of the RP group were higher than that of NP group (t = − 6.14, P < 0.01; t = − 6.14, P < 0.01) (**a**,**c**). *P < 0.05.

The dose distributions in the two groups are presented in Table 1 and Fig. 1. PTV in both groups that were irradiated at 100% of the prescribed dose approached 50 Gy. The V_RX_ of NP group (94.79 ± 0.93%) was higher than that of RP group (94.37 ± 0.88%) and was statistically significant (t = − 4.57, *P* < 0.01). The CI and HI of NP group were significantly higher than those of RP group. However, V_55_ (110% of the prescribed dose by volume) and D_mean_ of RP group were higher than those of NP group.

**Table 1:** Dosimetry comparison for the PTV of RP group and NP group (mean ± SD).

PTV	n	RP group	NP group	t	*P*
V_RX_	50	94.37 ± 0.88	94.79 ± 0.93	− 4.57	**0.00**^**a**^
CI	50	0.66 ± 0.05	0.72 ± 0.04	− 12.99	**0.00**^**a**^
HI	50	0.13 ± 0.02	0.1 ± 0.02	− 6.14	**0.00**^**b**^
V_55_	50	1.97 ± 1.82	0.19 ± 0.31	− 6.14	**0.00**^**b**^
Dmean	50	52.45 ± 0.33	51.9 ± 0.24	− 6.14	**0.00**^**b**^

*a = Paired t test, *b = Nonparametric Wilcoxon test, *P* < 0.05.

Bold values indicates that the difference is statistically significant (*P*<0.05)

### The improvement of dose to OAR

**Figure 2:**
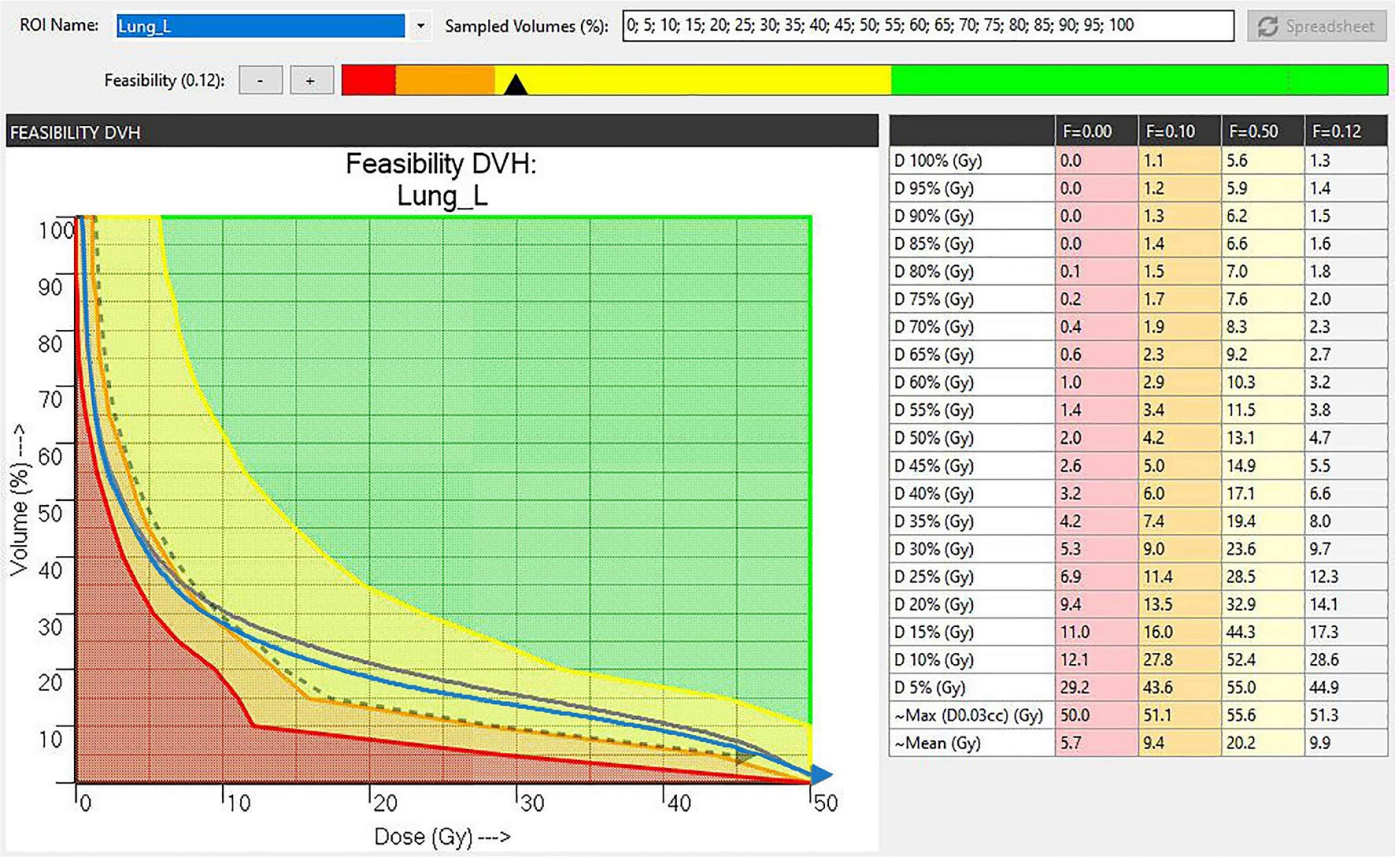
The FDVH diagram of ipsilateral lung. The green region was considered dosimetrically easy to achieve, yellow was more challenging, orange is difficult, and the red region indicates an region which cannot be achieved. The gray line represents the FDVH curve of the affected lung of NP group, while the dashed line represents the FDVH curve of the affected lung of RP group when F is 0.12. The V20 in the yellow region of great concern in RP group is significantly lower than that of NP group.

We chose ipsilateral lung of one patient as an example. In Fig. 2, we set F as 0.00, 0.10, 0.12, and 0.50, respectively, and marked the red, dark yellow, dashed, and light yellow lines. The area below each line represented the average dose to ipsilateral lung. The green region was considered easy to achieve; yellow was challenging; orange was difficult; red was hard to achieve if PTV was not sacrificed. The gray line represented the FDVH curve of the affected lung of NP group, whereas the dashed line represented that of RP group when F = 0.12. Compared with the two curves, V_20_ in the yellow region with great concern was lower in RP group than NP group.

The doses of the other OARs are shown in Table 2. In both groups, V_20_, V_10_ and D_mean_ in ipsilateral lung were lower in RP group than those in NP group. However, V_5_ of RP group was higher than that of NP group. All D_mean_ of the heart, breast-R; shoulder joint-L, trachea, esophagus, intestine in RP group were lower than those in NP group. Although D_mean_ of the thyroid was higher in RP group than in NP group, the difference was statistically insignificant. In addition, all D_max_ values of the spinal cord, shoulder joint-L, trachea, and intestines were lower in RP group than in NP group. However, D_max_ of the thyroid in RP group was higher than that in NP group. Although D_max_ of the esophagus in RP group was lower than that in NP group.

**Table 2:** Dosimetry comparison for the OAR of RP group and NP group (mean ± SD) (cGy).

OAR		n	RP group	NP group	t	*P*
Ipsilateral lung	V_20Gy_	50	19.56 ± 2.4	22.1 ± 2.06	− 6.15	**0.00**^**b**^
	V_10Gy_	50	31.01 ± 3.06	32.16 ± 2.84	− 4.20	**0.00**^**b**^
	V_5Gy_	50	45.83 ± 4.56	44.9 ± 3.48	2.38	**0.02**^**a**^
	D_mean_	50	10.68 ± 1.09	11.45 ± 1.02	− 6.14	**0.00**^**b**^
Heart	D_mean_	50	4.95 ± 1.72	5.38 ± 1.88	− 3.68	**0.01**^**a**^
Breast-R	D_mean_	50	11.19 ± 7.17	17.27 ± 12	− 4.79	**0.00**^**b**^
Spinal cord	D_max_	50	13.62 ± 3.16	22.67 ± 5.7	− 6.02	**0.00**^**b**^
shoulder joint-L	D_mean_	50	7.93 ± 2.2	12.38 ± 2.53	− 15.34	**0.00**^**a**^
	D_max_	50	20.06 ± 7.84	32.63 ± 7.74	− 13.71	**0.00**^**a**^
Thyroid	D_mean_	50	15.25 ± 4.82	14.6 ± 4.93	− 1.38	0.17^b^
	D_max_	50	47.26 ± 8.9	45.47 ± 7.89	− 3.66	**0.00**^**b**^
Trachea	D_mean_	50	6.79 ± 2.04	7.51 ± 3.25	− 2.48	**0.02**^**a**^
	D_max_	50	19.83 ± 8.46	22.85 ± 10.11	− 4.02	**0.00**^**a**^
Esophagus	D_mean_	50	3.94 ± 1.88	4.3 ± 2.55	− 1.68	**0.01**^**b**^
	D_max_	50	28.89 ± 12.76	30.78 ± 12.41	− 2.06	0.05^a^
Intestines	D_mean_	50	7.59 ± 5.85	12.46 ± 10.89	− 4.53	**0.00**^**b**^
	D_max_	50	32.58 ± 19.06	36.84 ± 18.78	− 2.15	**0.03**^**b**^

*a = Paired t test, *b = Nonparametric Wilcoxon test, *P* < 0.05.

Bold values indicates that the difference is statistically significant (*P*<0.05)

MU value of the IMRT plan designed in RP group was higher than that in NP group. The data are presented in Table 3.

**Table 3:** Dosimetry comparison for the MU of RP group and RP group (mean ± SD).

Group	n	MU
RP group	50	899.7 ± 120.89
NP group	50	752.34 ± 111.43
t		10.65
*P*		**0.00**^**a**^

*a = Paired t test, *P* < 0.05.

Bold values indicates that the difference is statistically significant (*P*<0.05)

### Scores of plans

We quantitatively evaluated PTV and OAR of all patients (Table 4). OAR scores of RP group was higher than that of NP group, whereas PTV scores of RP group was inferior to that of NP group. In both groups, the comprehensive OAR and PTV scores of NP group (107.00 ± 13.13) were higher than those of RP group (104.37 ± 12.64) with statistical significance.

**Table 4:** Evaluation comparison for the score of RP group and NP group (mean ± SD).

Structure	NP group	RP group	*t*	*P*
Total score	107.00 ± 13.13	104.37 ± 12.64	− 2.486	**0.013**^**a**^
PTV score	47.99 ± 3.91	37.72 ± 6.52	− 6.144	**0.000**^**a**^
OAR score	59.01 ± 11.41	66.65 ± 8.10	− 6.028	**0.000**^**a**^

*a = Paired t test, Wilcoxon test, *P* < 0.05.

Bold values indicates that the difference is statistically significant (*P*<0.05)

## Discussion

Breast cancer occurring ranks first among female malignant tumors^16,17^. RT can reduce the local recurrence rate and improve the effective survival rate of patients with high-risk factors^18,19^. The application of IMRT allows the postoperative tumor bed area to receive an adequate dose of preventive radiation and reduce radiation to OAR. Many researchers have studied the application of Plan-IQ in RT planning design and pointed out that it further protected OAR, which was also confirmed in our study^13–15,20^. Some researchers have used PQM on the dosimetric parameters of the entire RT plan to quantitatively evaluate the change in the RT plan quality from a macroscopic perspective^21–25^. However, no study has separated the PQM scores into PTV and OAR parts to evaluate IMRT planning after modified radical mastectomy. To quantitatively evaluate the quality of IMRT plans in the two groups, we scored PTV and OAR and allocated each measure maximum scores of 20 according to the rating scale we set up.

OAR dose limits of the 50 cases of IMRT plan fully met the requirements of the RTOG and NCCN guidelines. We optimized the dose limit of OAR by referring to the FDVH. The D_mean_, V_20,_ and V_10_ of ipsilateral lung in RP group were significantly improved than those in NP group, including a decrease of about 2.0% in V_20_ and approximately 1 Gy in D_mean_. In the manual planning design, it was difficult for V_20_ to exhibit a downward trend. However, our study showed that Plan-IQ could reduce V_20_ while ensuring the prescribed dose of PTV and the safety of other OARs. However, the value of V_5_ in RP group was approximately 1% higher than that in NP group. Clinically, V_20_ and D_mean_ are decisive factors in radiation pneumonia occurrence^26,27^. The increase in V_5_ was less than 1%, whereas the significant decrease in V_20_ and D_mean_ may bring more incredible benefits to the patients^28,29^.

The NCCN guidelines clearly indicate that the incidence of radiation-induced coronary heart disease can be reduced when D_mean_ of the heart is below 8 Gy^30^. In our study, D_mean_ of the heart in RP group was below 7 Gy. Other OARs, such as the esophagus, main trachea, stomach, and intestine, with smaller irradiated volume, easily were ignored during design planning, but Plan-IQ accounted for all OARs. The protection in RP group for the breast-R, spinal cord, humeral head, trachea, esophagus, and intestines was apparently better than that in NP group. However, RP group did not show an advantage in the protection of the thyroid, possibly because we only emphasized the single parameter F. The dose of OAR in RP group was lower than that in NP group, but the coincidence degree with the predicted result of Plan-IQ was not perfect. This was mainly because Plan-IQ did not consider the type of planning system, the intensity modulation mode (IMRT or VMAT), and the multi-leaf collimator thickness when predicting the DVH of OAR. Although there were some shortcomings, Plan-IQ provided a significant improvement in OAR, which offered a new way for the physicist to solve the problem of the plan design. In addition, the optimized process of OAR inevitably led to longer treatment durations, which were reflected in an increase in MU and submitted more elaborate planning in RP group.

In the PQM scores of PTV, the scores of the RP plan decreased compared with NP group. It can be clearly seen from Fig. 1 that the main scores losses are CI, HI, and V_55_. The results also showed that RP group had an advantage in OAR while reducing CI, HI, and V_55_ scores. As the dose of OAR decreased, the V_55_ increased from 0.19% to 1.98%. In contrast, although OAR scores in NP group was not as high as that of RP group, its scores in PTV maintained an advantage. However, the difference in total scores between the two groups was only approximately 3 points. Although the scores decreased, V_RX_, the main parameter for evaluating PTV dosimetry, did not change significantly, and all PTV indicators were fully satisfied with the clinical requirements.

In this study, although the total scores of RP group did not improve, the individual scores of PTV and OAR were significantly changed. PTV scores in RP group dropped, but OAR scores improved more. The scores loss of PTV on CI, HI, and V_55_ could be ignored compared with the protection of OAR. Our results also indicated that if we want to improve OAR further, we have to sacrifice the CI, HI, and V_55_ of PTV. The introduction of Plan-IQ in the IMRT plan contributed to predicting the remaining reduction space of OAR dose limit, especially the V_20_ and D_mean_ of the affected ipsilateral lung. Although the results came at the expense of CI, HI, and V_55_ of PTV, it was still very beneficial to patients.

## Conclusion

In conclusion, in the RT process after modified radical mastectomy, Plan-IQ can automatically help physicians design the best OAR protection plan, which sacrifices part of PTV, but still meets the clinical requirements. If further breakthroughs are to be made, clinicians need to decide on the balance between PTV and OAR to determine the next goal of the RT plan design. The plan quality scoring added an evaluation measure that quantified the plan quality of individual patients.

## Supplementary Information

Supplementary Table S1.

## Data Availability

We confirmed that all data were obtained from the First Hospital of Jilin University. Research data are stored in an institutional repository and will be shared upon request to the corresponding author.
