# Supplementary material for: Systematic quantitative evaluation of Plan-IQ for intensity-modulated radiation therapy after modified radical mastectomy
Source: Sci Rep. 2021 Nov 8;11:21879. doi: 10.1038/s41598-021-01305-3 (PMC8575920; doi:10.1038/s41598-021-01305-3)
Supplement: Supplementary file 1 — Supplementary Table S1. [file 41598_2021_1305_MOESM1_ESM.docx]

Table S1. IMRT radiotherapy plan score scale

| **Target** | [**Structure**](C:/Users/Administrator/AppData/Local/youdao/dict/Application/8.9.6.0/resultui/html/index.html#/javascript:;) | **Indicators** | **Unit** | **Disqualification** | | **Aualification** | | **Excellence** | | **Perfect** | |
| --- | --- | --- | --- | --- | --- | --- | --- | --- | --- | --- | --- |
|  |  |  |  | **Evaluation** | **Score** | **Evaluation** | **Score** | **Evaluation** | **Score** | **Evaluation** | **Score** |
| **PTV** | PTV | V50 | % | ＜92 | -50 | ≥92 | 15~18 | ≥98 | 18~20 | 100 | 20 |
|  | PTV | V55 | % | ≥3 | 0 | ＜3 | 0~1 | ≤2 | 1~10 | 0 | 10 |
|  | PTV | HI | / | - | - | ≤0.3 | 0~ | ≤0.1 | 9~10 | 0 | 10 |
|  | PTV | D50 | Gy | - | - | ≤55 | 0~5 | ≤52 | 5~10 | 50 | 10 |
|  | PTV | CI | / | - | - | ≥0.5 | 0~9 | ≥0.7 | 9~10 | 1 | 10 |
| **OAR** | LUNG_L | V20 | % | ≥30 | -50 | ≤25 | 0~8 | ≤20 | 8~10 | ≤15 | 10 |
|  | LUNG_L | V10 | % | - | - | ≤40 | 0~3 | ≤35 | 3~5 | ≤30 | 5 |
|  | LUNG_L | V5 | % | - | - | ≤60 | 0~3 | ≤55 | 3~5 | ≤50 | 5 |
|  | LUNG_L | Dmean | Gy | ≥13 | -50 | ＜13 | 0~3 | ≤12 | 3~5 | ≤10 | 5 |
|  | shoulder joint-L jiont[**shoulder**](C:/Users/Administrator/AppData/Local/youdao/dict/Application/8.9.6.0/resultui/html/index.html#/javascript:;) **[joint](C:/Users/Administrator/AppData/Local/youdao/dict/Application/8.9.6.0/resultui/html/index.html#/javascript:;)**joint | Dmean | Gy | - | - | ≤16 | 0~6 | ≤14 | 6~8 | ≤10 | 8 |
|  | shoulder joint-L | Dmax | Gy | - | - | ≤20 | 0~1 | ≤18 | 1~2 | ≤10 | 2 |
|  | heart | Dmean | Gy | - | - | ≤9 | 0~2 | ≤8 | 2~8 | ≤7 | 8 |
|  | heart | Dmax | Gy | - | - | ≤53 | 0~2 | - | - | ≤40 | 2 |
|  | thyroid | Dmean | Gy | - | - | ≤17 | 0~2 | ≤15 | 2~8 | ≤8 | 8 |
|  | thyroid | Dmax | Gy | - | - | ≤53 | 0~2 | - | - | ≤40 | 2 |
|  | breast_R | Dmax | Gy | - | - | ≤15 | 0~2 | - | - | ≤8 | 5 |
|  | intestines | Dmean | Gy | - | - | ≤20 | 0~2 | - | - | 0 | 2 |
|  | intestines | Dmax | Gy | - | - | ≤53 | 0~3 | - | - | ≤10 | 3 |
|  | trachea | Dmean | Gy | - | - | ≤10 | 0~3 | - | - | ≤2 | 2 |
|  | trachea | Dmax | Gy | - | - | ≤55 | 0~2 | - | - | ≤20 | 3 |
|  | esophagus | Dmean | Gy | - | - | ≤30 | 0~2 | - | - | 0 | 2 |
|  | esophagus | Dmax | Gy | - | - | ≤53 | 0~2 | - | - | ≤40 | 3 |
|  | spinal cord | Dmax | Gy | ﹥45 | -50 | ≤35 | 0~10 | 27 | 8~10 | ≤25 | 10 |
